# Supplementary figures and images for: A comprehensive PDCoV-host proteome interaction map reveals potential antiviral targets
Source: PLoS Pathog. 2025 Oct 24;21(10):e1013615. doi: 10.1371/journal.ppat.1013615 (PMC12551863; doi:10.1371/journal.ppat.1013615)

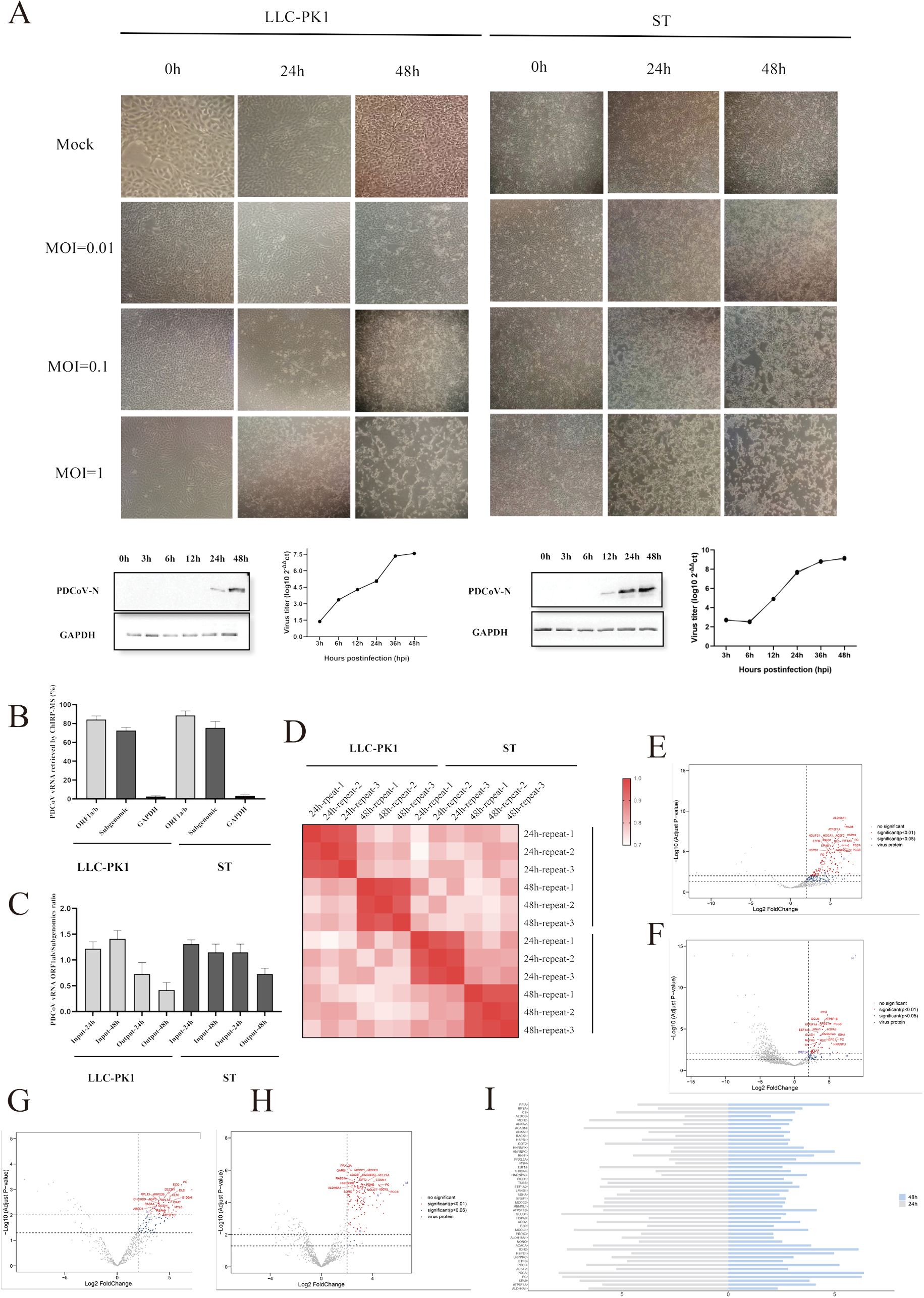

Supplement: S1 Fig — (B) vRNA recovery efficiency by ChIRP-MS protocol. (C) Percentage distribution of genomic (ORF1a/b) vs. subgenomic RNA reads pre-/post-pulldown. (D) Inter-sample correlation coefficients (n = 3) at 24/48hpi in ST/PK1 cells. (E)-(H) Host binding proteins identified in LLC-PK1/ST cells at 24/48hpi. (I) Persistent host interactors across timepoints in PK1 cells. (TIF) [file ppat.1013615.s001.tif]

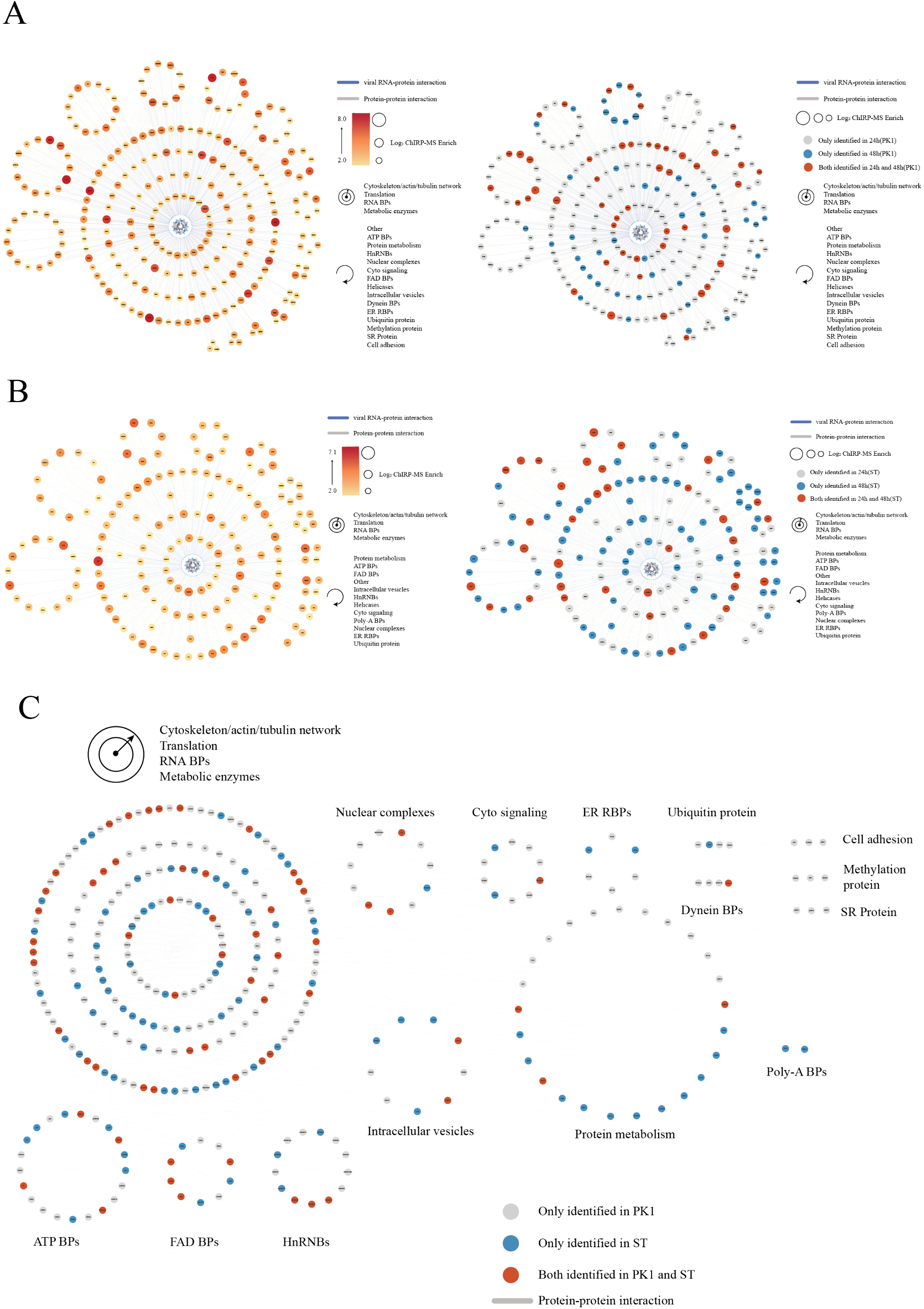

Supplement: S2 Fig — Right: Pathway preferences at different infection stages. (B) Left: Host interaction network in ST cells. Right: Temporal pathway enrichment. (C) Cell line-specific differences in PDCoV-host protein interactions. (TIF) [file ppat.1013615.s002.tif]

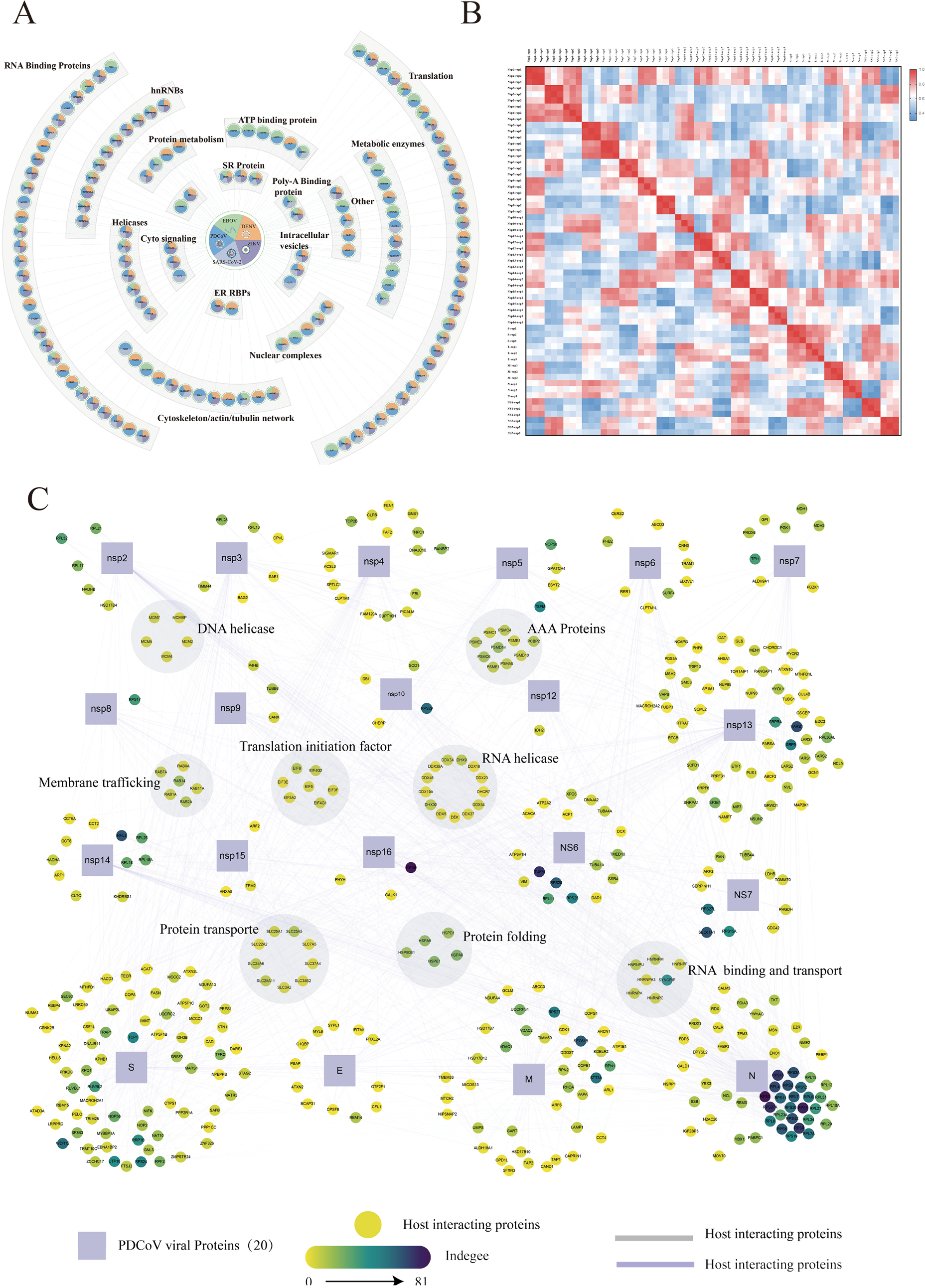

Supplement: S3 Fig — (B) Inter-replicate correlation coefficients (n = 3) for AP-MS-identified vProtein interactomes. (C) Cytoscape network of vProtein-host interactions (square nodes: viral proteins; color gradient by Indegree value; gray clusters: cellular pathways). (TIF) [file ppat.1013615.s003.tif]

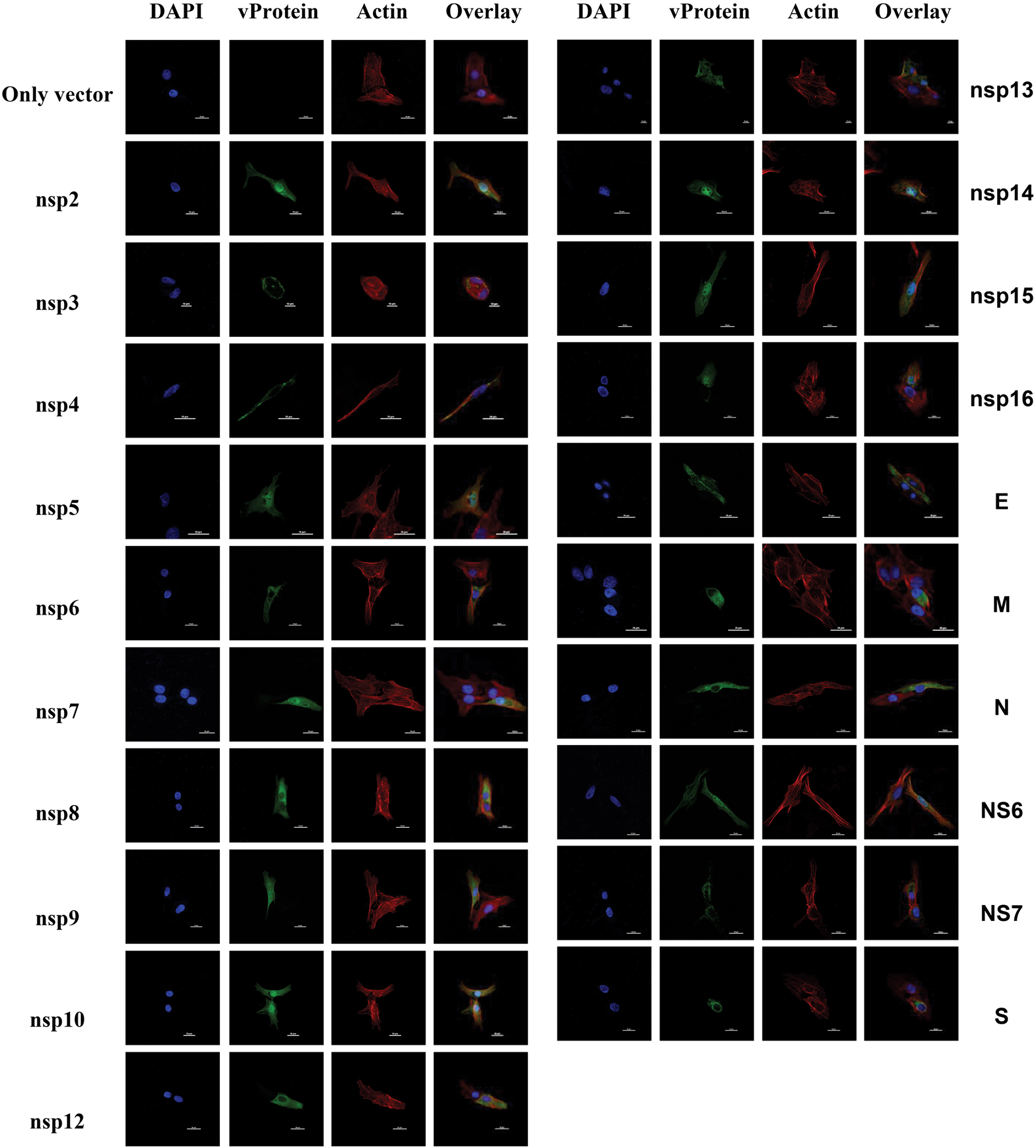

Supplement: S4 Fig — (TIF) [file ppat.1013615.s004.tif]

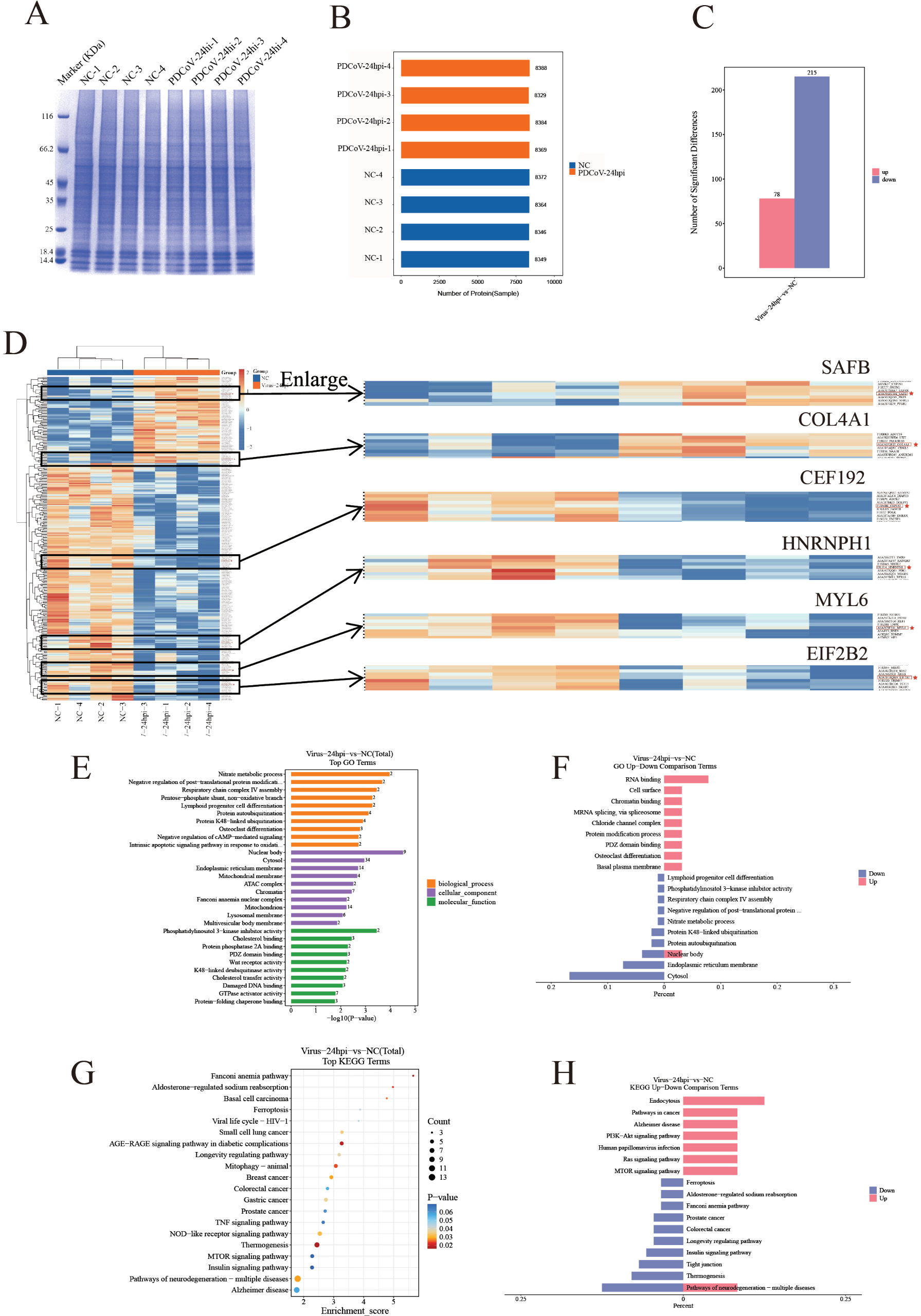

Supplement: S5 Fig — (B) Number of protein hits in 4D-DIA proteomics samples. (C) Identification of differentially expressed host proteins (P-value < 0.05, FC ≥ 1.2 or FC ≤ 1/1.2) in PDCoV-infected PK1 cells. (D) Left: Heatmap clustering of DEPs expression levels (red: high; blue: low). Right: Expression changes of six PDCoV-interacting host proteins pre-/post-infection. (E) Top GO terms for DEPs (-log10 p-value vs. term; bar labels indicate protein counts). (F) Comparative GO enrichment (top 10 up/down-regulated terms by p-value; x-axis: ListHits/TotalHits ratio). (G) KEGG pathway analysis of DEPs (bubble size: protein count; color gradient: p-value significance). (H) Comparative KEGG enrichment (top 10 up/down-regulated pathways by p-value). (TIF) [file ppat.1013615.s005.tif]

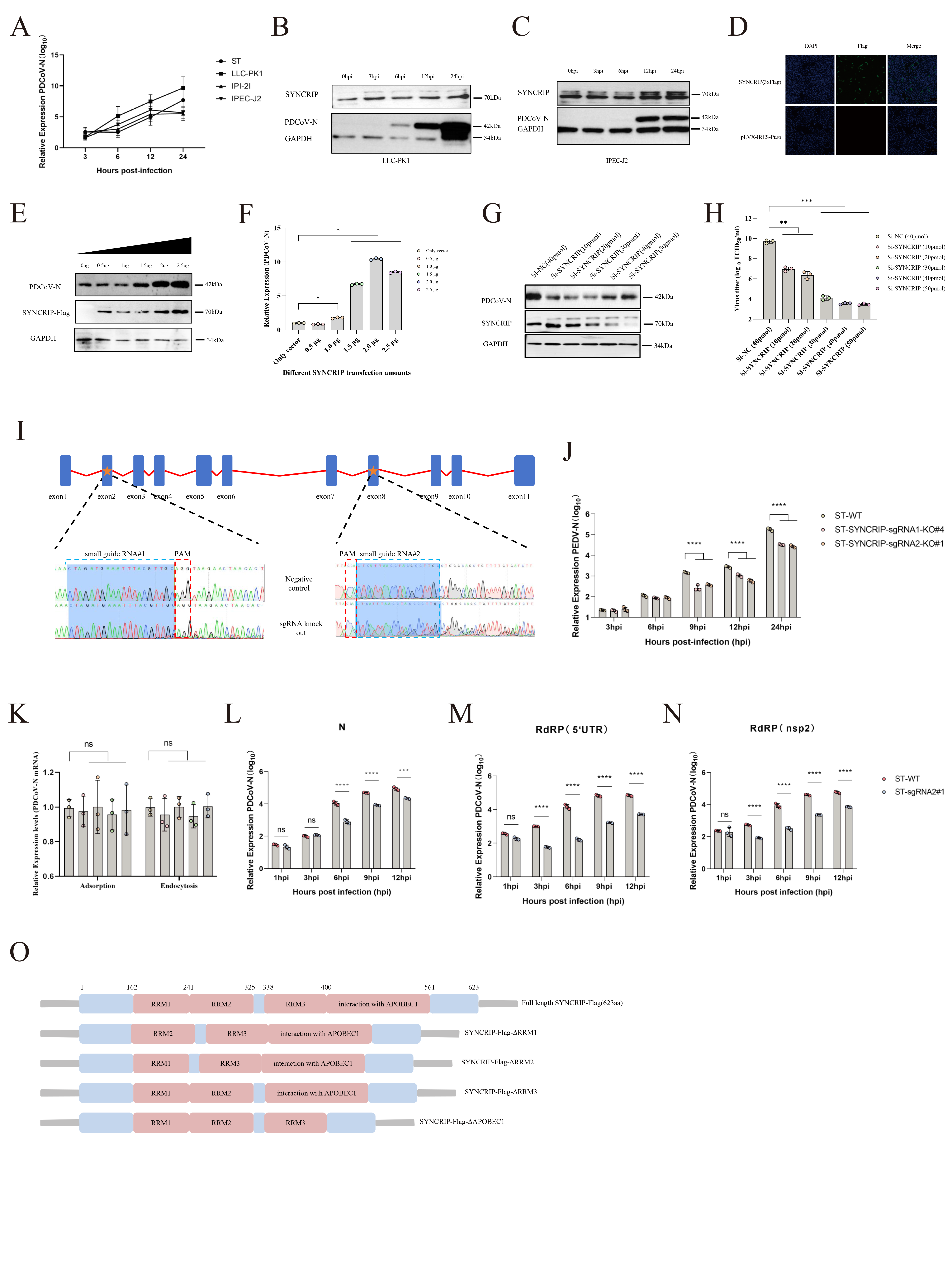

Supplement: S6 Fig — (B) Temporal SYNCRIP protein expression in PDCoV-infected LLC-PK1 cells. (C) SYNCRIP expression dynamics in infected IPEC-J2 cells. (D) IFA validation of SYNCRIP (Flag-tagged) expression. (E) Dose-dependent effect of SYNCRIP overexpression on viral N protein in IPEC-J2 cells. (F) Impact of SYNCRIP overexpression on viral mRNA replication. (G) Viral protein suppression by siRNA-SYNCRIP-2# treatment (gradient concentrations). (H) Dose-responsive inhibition of viral mRNA by siRNA-SYNCRIP-2#. (I) Sanger sequencing confirmation of exon2/exon-targeting sgRNA knock-in. (J) SYNCRIP knockout significantly inhibits PEDV mRNA replication. (K) Effect of SYNCRIP knockout on viral attachment/entry phases. (L)-(N) Impact on PDCoV N/RdRP(5’UTR)/RdRP(nsp2) mRNA replication in knockout cells. (O) Domain architecture of SYNCRIP and deletion mutant schematics. (TIF) [file ppat.1013615.s006.tif]

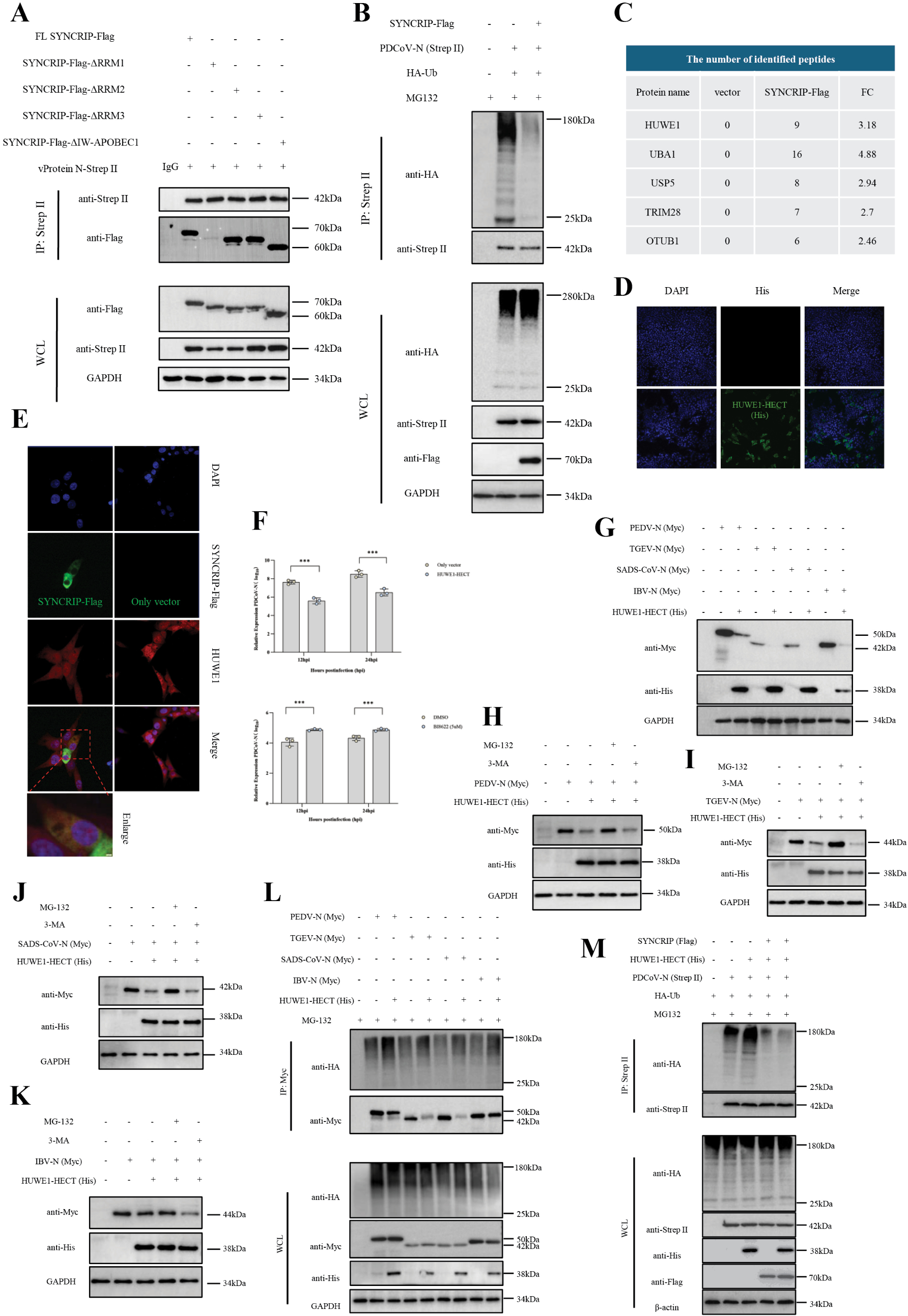

Supplement: S7 Fig — (B) Co-IP analysis of PDCoV N ubiquitination status following SYNCRIP overexpression. (C) IP-MS identification of host ubiquitination machinery components interacting with SYNCRIP. (D) IFA verification of HUWE1-HECT (His-tagged) expression in ST cells. (E) Confocal colocalization of SYNCRIP (Flag-tagged) with endogenous HUWE1. (F) Top: Impact of HUWE1-HECT overexpression on viral replication. Bottom: Effect of HUWE1 inhibitor BI8622 on PDCoV N mRNA replication. (G) Effect of HUWE1-HECT overexpression on PEDV/TGEV/SADS-CoV/IBV N protein levels. (H)-(K) WB analysis of N protein stability under proteasome/lysosome inhibition. (L) Ubiquitination status of other coronavirus N proteins with HUWE1-HECT overexpression. (M) Co-IP assay was performed to examine the ubiquitination level of PDCoV N protein mediated by HUWE1-HECT domain upon SYNCRIP overexpression. (TIF) [file ppat.1013615.s007.tif]

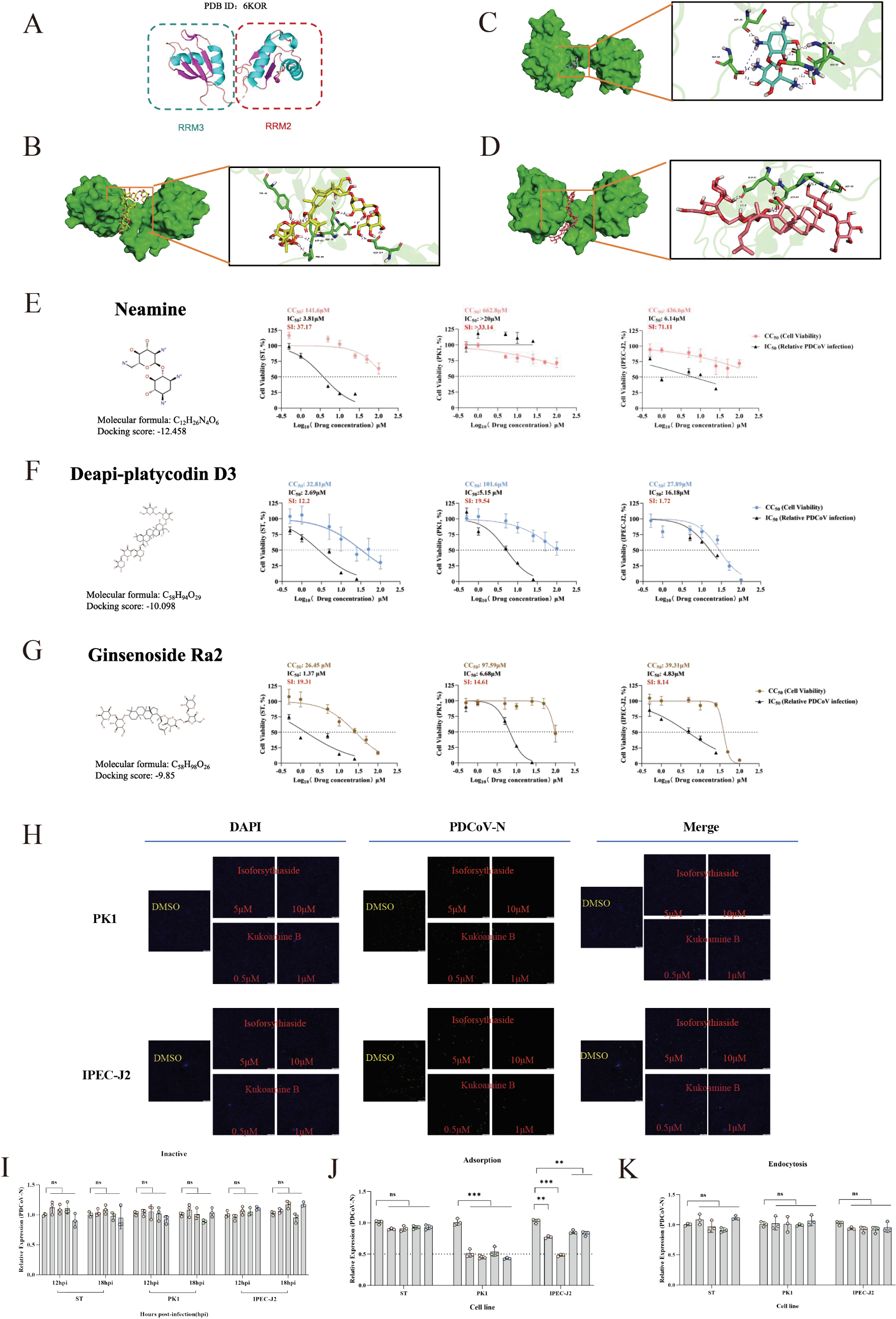

Supplement: S8 Fig — (B)-(D) Molecular docking models of Neamine/Deapi-platycodin D3/Ginsenoside Ra2 with SYNCRIP. (E)-(G) CC50/IC50 determination for three compounds in PK1/ST/IPEC-J2 cells. (H) IFA quantification of PDCoV N suppression by compounds in PK1/IPEC-J2 cells. (I)-(K) Stage-specific inhibition of viral inactivation/attachment/entry by treatments. (TIF) [file ppat.1013615.s008.tif]
