# Supplementary material for: A comprehensive PDCoV-host proteome interaction map reveals potential antiviral targets
Source: PLoS Pathog. 2025 Oct 24;21(10):e1013615. doi: 10.1371/journal.ppat.1013615 (PMC12551863; doi:10.1371/journal.ppat.1013615)
Supplement: Attachment — Submitted filename: Response to Reviewers.docx [file ppat.1013615.s016.docx]

Dear Editors,

Thank you for your letter concerning our manuscript entitled “A comprehensive PDCoV-host proteome interaction map reveals potential antiviral targets” (ID: PPATHOGENS-D-25-01649). Those comments are all valuable and very helpful for revising and improving our paper, as well as the important guiding significance to our researches. We have studied comments carefully and have made correction which we hope meet with approval. Revised portion are marked in yellow in the manuscript. The main corrections in the paper and the responds to the reviewer’s comments are as following:

**Editor comment:**

**Question:** None of the figures in this manuscript are legible when printed - the font is illegibly small. The authors must correct the figures to be legible when printed on standard paper and then re-submit for further consideration.

**Response**: In response to the previous feedback regarding the small font size in our figures being difficult to read, we have carefully revised all figures by adjusting both the font sizes and overall dimensions. We have also streamlined some images to enhance the clarity of the remaining figures. Previous Section Editor: Michael Letko, PhD

Thank you for your careful review. We really appreciate your efforts in reviewing our manuscript during this unprecedented and challenging time. We wish good health to you, your family, and community. Your careful review has helped to make our study clearer and more comprehensive.

Once again, thank you very much for your comments and suggestions

Yours Sincerely,

Xin Yang
