# Supplementary material for: A comprehensive PDCoV-host proteome interaction map reveals potential antiviral targets
Source: PLoS Pathog. 2025 Oct 24;21(10):e1013615. doi: 10.1371/journal.ppat.1013615 (PMC12551863; doi:10.1371/journal.ppat.1013615)
Supplement: Attachment — Submitted filename: Response_to_Reviewers_auresp_1.docx [file ppat.1013615.s017.docx]

Dear Editors and Reviewers:

Thank you for your letter and for the reviewer’s comments concerning our manuscript entitled “A comprehensive PDCoV-host proteome interaction map reveals potential antiviral targets” (ID: PPATHOGENS-D-25-01700). Those comments are all valuable and very helpful for revising and improving our paper, as well as the important guiding significance to our researches. We have studied comments carefully and have made correction which we hope meet with approval. Revised portion are marked in red in the manuscript. The main corrections in the paper and the responds to the reviewer’s comments are as following:

**Reviewers comment:**

**Reviewer #1:**

**Q1:** While the interaction between SYNCRIP and N proteins is well-documented, the exact molecular mechanism by which SYNCRIP prevents HUWE1-mediated ubiquitination could be further explored. Structural studies or detailed mutagenesis analysis might strengthen this section.

Response: While current research lacks direct evidence on the interaction between SYNCRIP and HUWE1, and SYNCRIP itself has not been sufficiently studied in disease or pathogen contexts, we acknowledge that the 3D-modeled structure of SYNCRIP may significantly differ from its native form. This limitation currently prevents us from structurally investigating how SYNCRIP facilitates HUWE1's recognition of PDCoV N protein. However, we have provided new insights in lines 552-554 and Figure S7M Fig, demonstrating that SYNCRIP overexpression substantially impairs HUWE1-HECT-mediated ubiquitination of PDCoV N protein. To further elucidate this mechanism, we propose to determine the crystal structure of SYNCRIP. We are confident that precise structural data will significantly advance our understanding of SYNCRIP's role in coronavirus infection pathways.

**Q2:** The in vivo piglet study with Isoforsythiaside shows promise, but additional pharmacokinetic/pharmacodynamic data would strengthen the therapeutic potential claim.

Response: We believe comprehensive pharmacokinetic/pharmacodynamic data are crucial for evaluating the antiviral efficacy of Isoforsythiaside. While this study preliminarily investigated its in vivo inhibitory effects on PDCoV with limited data, the results clearly demonstrate its significant antiviral potential. However, we acknowledge that these findings are insufficient for definitive conclusions. Therefore, we are actively establishing a more robust evaluation system to assess Isoforsythiaside's antiviral properties. This includes dose optimization, administration method comparison, therapeutic/preventive efficacy analysis, and in vivo inhibition studies against additional viruses (PEDV/IBV/PRRSV/PCV) to comprehensively evaluate its pan-antiviral potential. Moving forward, we prioritize pharmacokinetic/pharmacodynamic studies of Isoforsythiaside in swine models, aiming to provide compelling evidence of its antiviral effects for the broader research community.

**Q3:** The comparison with other coronavirus-host interactomes (Fig 2E) is somewhat superficial. A more detailed discussion of conserved vs. unique interaction patterns would be valuable.

Response: Previously, our focus was limited to these conserved host proteins. However, in lines 220-229, we have now included descriptions of PDCoV-specific RNA-binding proteins. Additionally, in the Discussion section (lines 729-740), we have expanded on the functional implications of these conserved and unique interaction patterns across different RNA viral infections.

**Q4:** Some quantitative proteomics data (e.g., spectral counts, peptide counts) are not fully reported, making it difficult to assess interaction confidence levels.

Response: In S5 Fig, we have added SDS-PAGE results and protein identification counts from the 4D-DIA proteomics samples. To prevent S5 Fig from becoming too crowded with additional panels, we have made adjustments to the figure layout by removing the previously included S5C/D/G/H panels. This modification improves the visual clarity of S5 Fig while ensuring all relevant figure legends and text descriptions are updated accordingly. Additionally, we have included detailed statistics on peptide and protein counts from the proteomics data in Supplementary Tab5.

**Q5:** The discussion could better integrate findings with current reports on coronavirus-host interactions.

Response: In the Discussion section (lines 717-723), we have supplemented reports on the interactions between other coronaviruses and host proteins. Additionally, we expanded the discussion in lines 695-704 regarding the discrepancies between the host interaction protein library generated in this study and those identified through CRISPR-Cas9-based host factor screening. While prior studies have investigated individual PDCoV-encoded proteins (e.g., S, N, nsp5, nsp12) and their interactions with host factors, comprehensive studies mapping global host interaction proteins across all PDCoV-encoded proteins or viral genomic RNA remain absent. Given that viral proteins often function cooperatively, analyzing individual viral-host interactions provides limited insights into how PDCoV collectively reprograms host cells. Therefore, this study aims to elucidate the complex network between PDCoV and its host, uncovering broader infection mechanisms.

**Reviewer #2:**

Introduction section‌

**Q1:** Could you supplement the applications of CHIRP-MS and AP-MS in screening host-interacting proteins for other viruses?

Response: We have added relevant studies on the application of CHIRP-MS and AP-MS techniques in screening virus-host interaction proteins, which can be found in lines 90-105.

**Q2:** Are there other studies reporting the combined use of CHIRP-MS and AP-MS on the same pathogen?

Response: To our knowledge, there are currently no reports on the combined use of CHIRP-MS and AP-MS for the same pathogen. We believe this integrated approach will provide significant insights into PDCoV infection mechanisms and prevention strategies.

**Q3:** Could you elaborate on SYNCRIP's functions and its role in viral infection?

Response: We have supplemented the research background on SYNCRIP functions in lines 111-117.

Results section‌

**Q4:** Viral RNA forms secondary structures that may affect the complementary pairing of labeled probes. How did you improve the binding efficiency between viral RNA and labeled probes? Did you test their binding efficiency?

Response: In this study, we used 4% formaldehyde (instead of UV) for crosslinking RNA-binding host proteins. Formaldehyde effectively opens RNA secondary structures and improves probe binding efficiency. While we were unable to directly test the binding efficiency between probes and viral RNA, previous studies reported >60% binding rates, which we consider sufficient for viral RNA recovery.

**Q5:** Line 146: How did you distinguish viral RNA from host RNA?

Response: We primarily distinguished viral RNA from host RNA through specifically designed primers: viral RNA was detected using ORF1ab-targeted primers, while host RNA was detected using GAPDH-targeted primers. This description has been added in lines 848-852.

**Q6**: Coronavirus sgRNAs are expressed unevenly. Did you test the proportions of each sgRNA in the recovered viral RNA?

Response: We sincerely apologize for not testing the proportions of various sgmRNAs in recovered viral RNA samples. We believe simple qPCR cannot accurately reflect the ratios of different sgmRNAs in "Input" and "Output" samples. In future studies screening host interaction proteins for other viral RNAs, we will perform RNA-seq analysis using Chip-seq technology.

**Q7:** Line 154: LC-MS detected signals from ORF1ab. What are the proportions of the nsps formed by ORF1ab cleavage?

Response: We identified mass spectrometry samples using MaxQuant software with two reference protein databases: Sus scrofa and PDCoV viral proteins (both downloaded from UniProt). However, due to limited research attention on PDCoV, the viral protein database lacks annotations for individual nsps, only including two polyproteins (pp1a and pp1b) and four structural proteins. Consequently, we couldn't analyze the content of each nsp in our samples.

**Q8:** Lines 188-206: I am particularly interested in the interaction features of the host-interacting proteins associated with the viral key proteins nsp5, nsp12, and S protein. Could you supplement these here?Response:

Response: We added descriptions about interaction proteome characteristics of three key viral proteins (nsp5, nsp12, and S) in lines 253-256.

**Q9:** Line 279: Did you use an interaction protein library from porcine-related viruses?

Response: This study represents the first comprehensive identification of host interaction proteins for both PDCoV genomic RNA and all encoded proteins. Particularly for genomic RNA, we found no similar studies in swine-related viruses. Although some studies have screened host interaction proteins for individual PDCoV proteins, viral protein interactions form a complex system where studying isolated proteins cannot fully reveal how PDCoV reprograms host cell processes during infection.

**Q10:** Line 321: Could you provide the binding status of these six significantly changed host proteins during infection with vRNA or vProtein?

Response: We added hit descriptions for six proteins in vRNA or vProtein interaction libraries in lines 376-380.

**Q11:** Line 356: Did you examine SYNCRIP's expression levels after in vivo viral infection?

Response: We attempted to evaluate SYNCRIP expression changes during viral infection using IHC. However, due to SYNCRIP's low expression in intestinal tissues, IHC appears unsuitable for detecting PDCoV-induced SYNCRIP expression changes.

**Q12:** Does PDCoV infection affect the localization of endogenous SYNCRIP?

Response: Previous studies haven't reported that SYNCRIP requires cellular localization changes to function. Our confocal microscopy observations confirmed that PDCoV infection doesn't alter endogenous SYNCRIP localization. Considering this negative result, we decided not to include it in our final results.

**Q13:** Line 373: Besides PEDV, did you test the replication of other porcine-related viruses in SYNCRIP-knockout cells?

Response: Our focus remains on porcine coronaviruses. The other two swine coronaviruses (TGEV and SADS-CoV) have extremely low prevalence, with SADS-CoV only found in limited regions. Currently lacking these viral materials, we plan future collaborations to investigate SYNCRIP's role in other viruses.

**Q14:** Fig 6A: Could you provide fluorescence localization signal images from confocal microscopy?

Response: We supplemented confocal fluorescence localization signal maps in Fig6A.

**Q15:** Line 449: What was the concentration of B18622 in PK1 cells?

Response: We treated PK1 cells with B18622 at concentrations of 1nmol, 10nmol, 50nmol, and 100nmol. CCK-8 assays showed >90% cell viability at 100nmol, which we subsequently used for all experiments. We added B18622 concentration details in line 513.

**Q16**: Line 469: Please list the amino acid differences of SYNCRIP proteins across species in a table.

Response: S6 Table summarizes SYNCRIP protein sequence variations across species.

**Q17:** Line 473: SYNCRIP interacts with the N protein of other coronaviruses. Can SYNCRIP similarly promote the replication of other coronaviruses?

Response: Due to experimental limitations, we currently cannot obtain other coronavirus strains. We will actively seek collaborations to explore SYNCRIP's role in other viruses.

**Q18:** Line 622: Could you explain the reasons for the low overlap between the receptors identified by CRISPR/Cas9 screening and this study's database?

Response: We expanded discussion about the limited overlap between CRISPR/Cas9 screening results and our database in lines 695-704.

**Q19**: Please standardize the spelling of "ChIRP-MS" throughout the manuscript.

Response: We standardized all "ChIRP-MS" terminology throughout the manuscript.

Thank you for your careful review. We really appreciate your efforts in reviewing our manuscript during this unprecedented and challenging time. We wish good health to you, your family, and community. Your careful review has helped to make our study clearer and more comprehensive.

Once again, thank you very much for your comments and suggestions

Yours Sincerely,

Xin Yang
